# Re-evaluation for systematic reviews of traditional Chinese medicine in the treatment of chronic bronchitis

**DOI:** 10.1097/MD.0000000000036472

**Published:** 2023-12-08

**Authors:** Yasheng Deng, Lanhua Xi, Siyin Han, Tianwei Liang, Hui Huang, Yanping Fan, Yiqing Zheng, Jiang Lin

**Affiliations:** a Guangxi University of Chinese Medicine, Nanning, China; b Hainan Provincial Hospital of Traditional Chinese Medicine, Haikou, China.

**Keywords:** chronic bronchitis, meta-analyses, systematic reviews, traditional Chinese medicine therapy

## Abstract

**Background:**

Chronic bronchitis (CB) is a common clinical chronic respiratory disease, which has a high incidence in the middle aged and elderly population. With the development of the disease, the number of acute attacks becomes more and more frequent, which leads to the continuous decrease of lung function. If not treated in time, it will lead to a variety of complications and seriously affect the quality of life of patients. Traditional Chinese medicine (TCM) or TCM combined with western medicine is highly effective in the treatment of CB disease. In recent years, there are many systematic reviews on the use of TCM therapy in the treatment of CB, and the efficacy and safety of TCM in the treatment of CB diseases are evaluated. The aim of this study was to re-evaluate the Meta analysis/Systematic reviews (MAs/SRs) of TCM for the treatment of CB, aiming to provide a clinical basis for the treatment of CB by TCM.

**Methods:**

Retrieval among Chinese and English databases such as China National Knowledge Infrastructure, Wanfang database, China Scientific Journals Database, SinoMed, PubMed, Web of Science, The Cochrane Library and EMbase, etc. were conducted within the duration from database establish Tion date to March 2023.The included research was independently conducted by 2 researchers for literature screening, data extraction, and quality evaluation. The AMSTAR 2 scale was used to evaluate the quality of the report, the PRISMA 2020 statement evaluated the quality of the report, the ROBIS tool evaluated the risk of bias, and the GRADE quality evaluation tool evaluated the quality of the evidence.

**Results:**

Fifteen MAs/SRs were included, for a total of 224 studies involving 20,710 patients with CB. The 15 studies included in AMSTAR 2 are of very low quality. The ROBIS evaluation results showed that 8 MAs/SRs were considered to have high risk and 7 with low risk. The PRISMA 2020 report quality showed evaluation results of the included studies scores between 24 and 30, among them 13 with high quality and 2 with low quality. The GRADE system results showed that, within 70 outcome indicators, only 14 of them have moderate quality for evidence, with 31 for low quality, 25 for very low quality, and none for high quality.

**Conclusion:**

The MAs/SRs methodological quality of using TCM for treatment CB is generally poor, the quality of reports as well as evidence are generally low, and the risk of bias is high, therefore we should treat these results with caution.

## 1. Introduction

Chronic bronchitis (CB) is a chronic nonspecific inflammatory disease that occurs in the trachea, bronchial mucosa and surrounding tissues. The clinical symptoms include mainly cough and phlegm, often accompanied by shortness of breath and poor breathing. The seizure of disease each year usually lasts for more than 3 months within 2 years or more.^[1,2]^ The prevalence rate of CB among people is 4%, mostly seen in the middle-aged and elderly people, and the prevalence rate of people over the age of 50 can be as high as 15%.^[3–5]^ It is characteristized with a long course of disease and repeated attacks. If not treated in time, it would easily increase the risk of suffering from lung infections, pulmonary heart disease, obstructive pulmonary disease, and heart failure.^[6,7]^ Recent studies have shown that the prevalence of CB is on the rise worldwide. The occurrence of this disease has a significant impact on the quality of life, families of patients, society and health system, and has become one of the important public health issues.^[8–11]^

At this stage, the pathogenesis of CB is not completely clear. Clinical scholars believe that its pathogenesis is related to infection, environmental air pollution, allergy factors, decreased immunity, physiologically anatomical structure of the bronchus and many other factors.^[12,13]^ At present, symptomatic treatments such as cough relieving, phlegm reduction, anti-infection, spasmolysis and asthma relieving are often used clinically for CB.^[14,15]^ Such drugs can effectively relieve patients’ symptoms and control the progress of the disease, but long-term use is prone to drug resistance and insensitivity, and the long-term effect is poor, so it is necessary to find reliable alternative treatment strategies. It is worth noting that traditional Chinese medicine (TCM) shows great advantage over others in the treatment of CB. A large number of studies have shown that TCM has a significant effect on the treatment of CB, and it has unique advantages in relieving patients’ symptoms, improving lung function, inhibiting inflammation, and regulating the body’s immunity.^[16–21]^

In recent years, a large number of MAs/SRs articles on the treatment of CB by TCM have been published. High-quality MAs/SRs can provide objective and reliable evidence and contribute to clinical practice. However, many studies have shown that the quality of existing MAs/SRs varies greatly. Due to its methodological and reporting defects, and insufficient evidence quality assessment, the conclusions drawn may be biased and may even affect medical decision-making. Therefore, it is essential to fully evaluate the quality of MAs/SRs. In this study, the AMSTAR 2 scale,^[22]^ PRISMA 2020 statement,^[23,24]^ ROBIS tool,^[25,26]^ GRADE system tool^[27]^ and other methods were used to comprehensively evaluate the methodological quality, risk of bias, reporting quality, evidence quality and efficacy of published TCM therapies as interventions for the systematic reviews of CB treatment, and summarize and evaluate the evidence of TCM for the treatment of CB, with a view to providing more scientific and reliable evidence support for the treatment of CB clinically, aiming to provide reference and ideas for future medical and health decision-making and the formulation of related guidelines.

## 2. Materials and methods

### 2.1. Study registration

The protocol of this Systematic reviews and Re-Evaluation was written and registration was performed using the official PROSPERO website. The registration number is CRD42023430363.

### 2.2. Search methods and strategies

**Figure 1.**
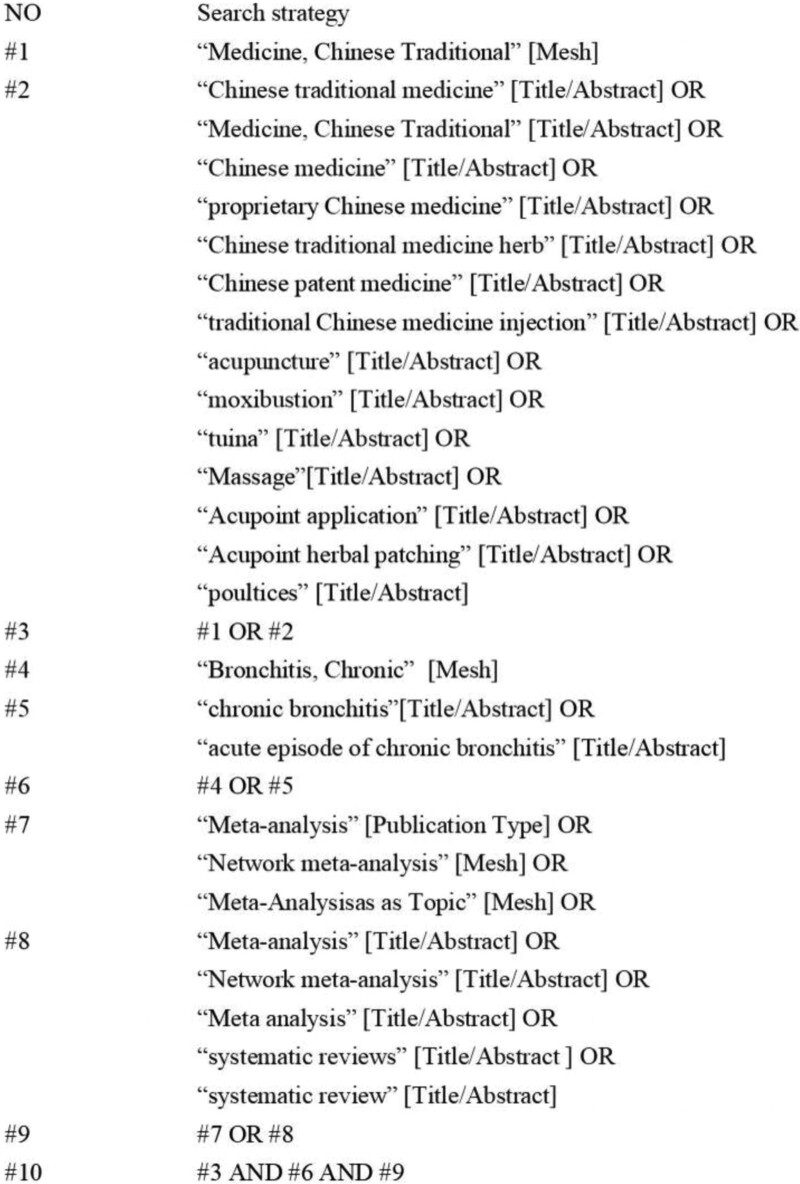
Pubmed search strategy.

Computer searches of the China National Knowledge Infrastructure, Wanfang Data, China Scientific Journals Database, SinoMed, PubMed, EMbase, and the Cochrane Library for literature on randomized controlled trials (RCTs) of CB treatment using TCM therapies in MAs/SRs, and the retrieval time limit is from the date of establishment of the library to March 2023. In addition, trace the references included in the literature, obtain the original text as much as possible, and search the gray literature to prevent missed inspections. The search method adopts a combination of keywords and free words, and the corresponding adjustments are made in combination with the search system. Search terms: chronic bronchitis, acute exacerbation of chronic bronchitis, traditional Chinese medicine, traditional Chinese medicine, proprietary Chinese medicine, traditional Chinese medicine injection, massage, acupoint application, Meta-analysis, systematic review, meta-analysis, etc. Taking PubMed retrieval as an example, the specific retrieval strategy is shown in Figure 1.

### 2.3. Research objects

The diagnostic criteria are in line with the definition and diagnostic criteria of the onset of CB in the “Practical Internal Medicine”^[28]^ and the “Guidelines for the Diagnosis and Treatment of Cough (2021),”^[29]^ and do not distinguish between age, gender, race, symptom type, length of course, and severity of the condition.

### 2.4. Intervention measures

Treatment group: Use TCM therapy methods (traditional Chinese medicine, proprietary Chinese medicine, TCM injection, acupuncture, tuina, acupoint application and other external treatments of TCM) or use TCM as the main combination of cough, phlegm, anti-infection and other Western medicine treatments. Control group: no treatment, placebo, Western medicine treatment, etc.

### 2.5. Outcome indicators

Including but not limited to the following indicators: efficiency, total efficiency, serum inflammatory factor level (interleukin [IL]-6, IL-8, C-reaction protein [CRP], tumor necrosis factor-α [TNF-α], etc.), clinical symptoms and signs disappearance time (cough disappearance time, cough and phlegm symptoms disappearance time, wheezing symptoms disappearance time, etc.), lung function indicators and blood gas analysis indicators (forced expiratory volume in one second [FEV1], forced vital capacity [FVC], FEV1/FVC, arterial oxygen partial pressure, etc.).

### 2.6. Exclusion criteria

Systematic reviews process included non-clinical RCTs studies; systematic reviews for which specific data were not available; duplicate published systematic reviews literature; and literature that did not include any of the primary outcome indicators.

### 2.7. Data extraction

In this paper, 2 researchers (L.X. and S.H.) independently searched and extracted the literature, while cross-checking the final screening results of both, and then independently extracted the included literature information according to a pre-designed data extraction form, with the assistance of a third researcher (T.L.) to judge in case of disagreement. The extracts included title, first author, publication date, journal of publication, study time span, number of included studies, sample size included, interventions and controls, outcome indicators, methodological quality assessment methods, risk of bias assessment tools, and main conclusions.

### 2.8. Quality assessment

Two researchers (H.H. and Y.Z.) evaluated the methodological quality, risk of bias assessment, quality of reporting and quality of evidence of the included literature through the AMSTAR 2 evaluation tool, ROBIS tool, PRISMA 2020 statement and GRADE system, which was checked by the researcher (Y.D.) after the evaluation was completed.

## 3. Results

### 3.1. Search results and basic characteristics of the included studies

**Figure 2.**
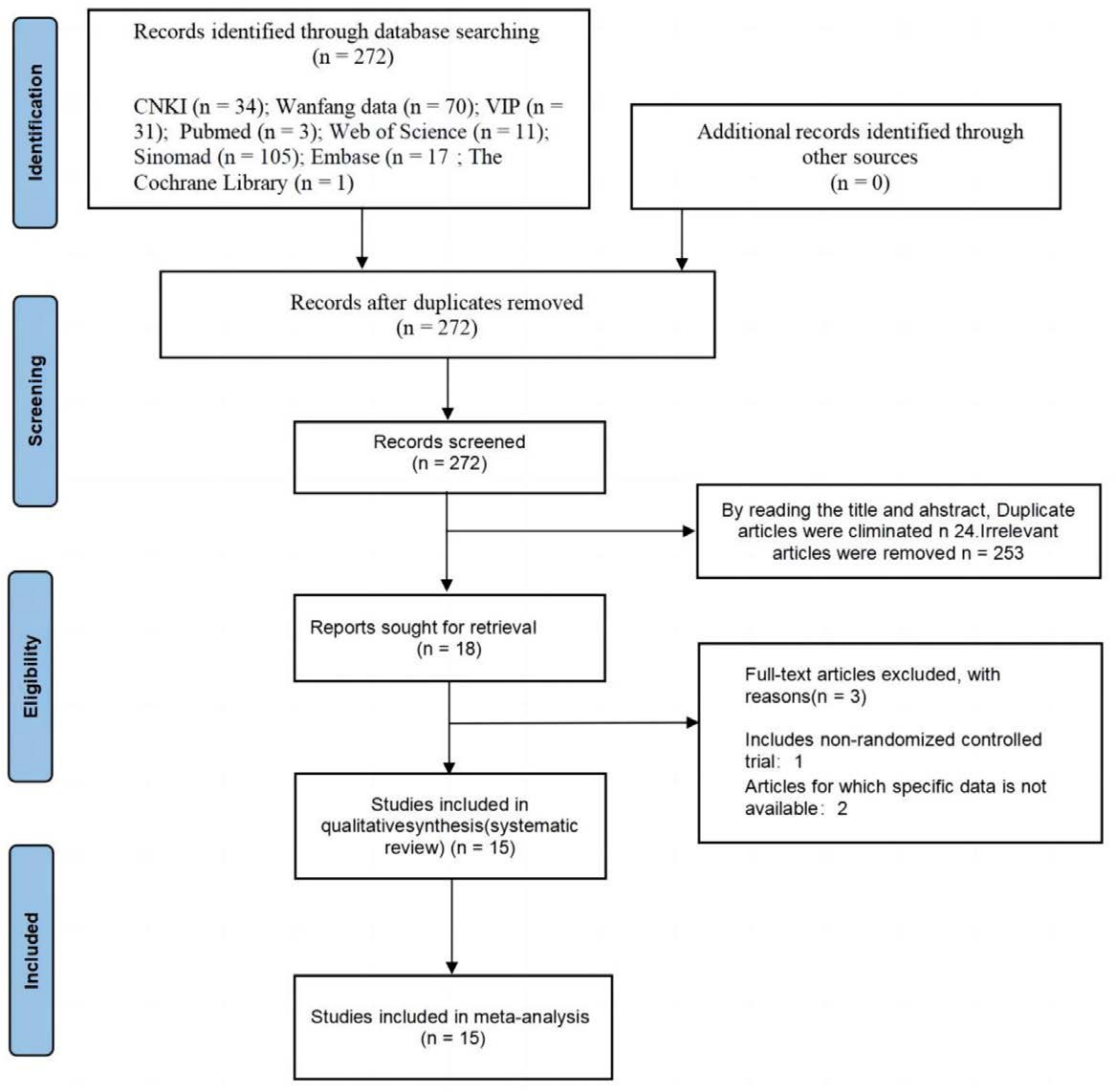
Flow chart of the study screening process.

By searching 8 databases, 272 articles were finally obtained. Thirty-four articles were retrieved from China National Knowledge Infrastructure, 70 articles were retrieved from WanFang Data, 31 articles were retrieved from China Scientific Journals Database, 3 articles were retrieved from Pubmed, 11 articles were retrieved from Web of science, and 105 articles were retrieved from Sinomad; 17 articles were retrieved from Embase database; 1 article was retrieved from the Cochrane Library Database. By reading the title of the article, 253 irrelevant and duplicate articles were excluded. By reading the full text of the article, 1 article containing non-RCTs and 2 articles with incomplete data were excluded. Included in 15^[30–44]^ SRs/Mas are journal literature, involving a total of 224 studies, including 20,710 patients, with a time span of 2013 to 2023.The specific search and filtering process is shown in Figure 2. The basic characteristics included in the study are shown in Table 1.

**Table 1:** Basic characteristics of the included studies.

Study	Types of included studies	Number of included studies	Sample size	Outcome indicators	Intervention measures		Methodology evaluation tools
					Therapy group	Control group	
Zhang et al 2023^[30]^	RCT	10	716	1b, 1e, 2a, 5a, 6	TCM/TCM+WM	WM	Cochrane
Li et al 2021^[31]^	RCT	5	535	1a, 2a , 4a	TCM+WM	WM	Cochrane
Liu et al 2021^[32]^	RCT	19	1517	1b, 2c, 2d, 2f, 3b, 3c, 3d, 3e	TCM+WM	WM	Cochrane
Mo et al 2021^[33]^	RCT	48	4356	1b, 3f, 6	TCM+WM	WM	Cochrane
Liu et al 2020^[34]^	RCT	12	1088	1b, 1d, 4d, 3a, 5b, 6	TCM/TCM+WM	WM	Cochrane
Ji et al 2016^[35]^	RCT	11	1169	1b, 2a, 1f, 6	TCM/TCM+WM	WM	Cochrane
Sun et al 201^[36]^	RCT	13	1556	1b	TCM	WM	Jadad
Tian. 2019^[37]^	RCT	5	612	1b, 5b, 6	TCM/TCM+WM	TCM/WM	Jadad
Zhu et al 2017^[38]^	RCT	19	1996	1b	TCM+WM	WM	Jadad
Dou et al 2022^[39]^	RCT	8	680	1b, 3f, 4a, 3d, 3e, 6	TCM+WM	WM	Cochrane
Gao et al 2019^[40]^	RCT	23	1901	1a, 2c, 2d, 2h, 6	TCM+WM	WM	Cochrane
Bai et al 2013^[41]^	RCT	14	1232	1b, 1c, 2h, 2c, 2e, 3f, 3h, 3j, 5b, 6	TCM+WM	WM	Cochrane
Zang et al 2021^[42]^	RCT	14	1147	1b, 2c, 2d, 2e	TCM+WM	WM	Jadad
Chu et al 2022^[43]^	RCT	10	956	2c, 2d, 2e, 2g, 2h, 3d, 3e, 3f, 3g, 3i, 4b, 4c, 4d, 4e, 4f, 4g	TCM+WM	WM	Cochrane
Liu et al 2017^[44]^	RCT	13	1249	1a, 2c, 2d, 2e, 6	TCM+WM	WM	Cochrane

RCT = randomized controlled trial, TCM = traditional Chinese medicine, WM = Western medicine.

1a total efficiency; 1b efficiency; 1c significant efficiency; 1d cure rate; 1e recurrence rate; 1f hospitalization time; 2a, clinical symptom disappearance/remission time; 2b dyspnea disappearance time; 2c cough disappearance/remission time; 2d cough and phlegm disappearance/remission time; 2e wheezing symptom disappearance/remission time; 2f chest inflammation disappearance time; 2g longyin disappearance time; 2h antipyretic time; 3a blood routine; 3b Hs-crp level; 3c IL-6 level; 3d IL-8 level; 3e TNF-α level; 3f CRP level; 3g WBC level; 3h neutrophil level; 3i PCT; 3j bacterial clearance rate; 4a lung function; 4b FEV1; 4c FVC; 4d FEV1/FVC; 4e PEF; 4f PaO2; 4g PaCO2; 5a symptom points; 5b TCM syndrome points; 6 adverse reactions.

### 3.2. Methodological quality assessment of the included studies

**Figure 3.**
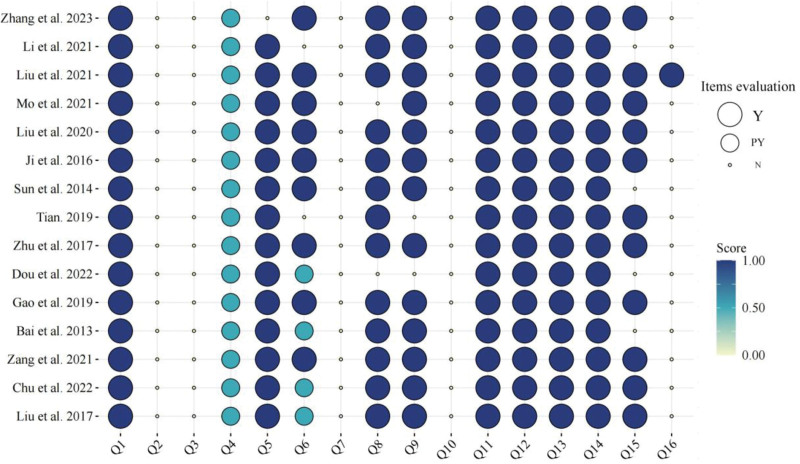
AMSTAR 2 Methodological quality assessment. Note: Y: yes; N: no; PY: partially yes. Items: Q1 the detail level of the research questions and inclusion criteria; Q2 the presence of a pre-design programmed; Q3 the presence of a rationale for the inclusion of study types; Q4 the comprehensiveness of the search strategy; Q5 whether the literature selection was double-repeat; Q6 whether the data extraction was double-repeat; Q7 the presence of a list of excluded literature and reasons for exclusion; Q8 whether the description of the included studies was detailed; Q9 whether the bias risk was taken with an appropriate tool; Q10 whether the funding source of individual studies was clear; Q11 whether the study results were combined using appropriate statistical methods; Q12 whether the impact of the bias risk of the included studies on the results was assessed; Q13 whether the bias risk of the included studies was considered; Q14 whether the heterogeneity of the study results was assessed; Q15 whether the possibility of publication bias was evaluated; Q16 whether relevant conflicts of interest were reported.

The AMSTAR 2 scale was used to evaluate the methodological quality of the 15 included studies. The literature credibility score was between 7 and 11.5, and all the included studies were rated as very low quality. Among them, 0 studies reported key items 2 and 7, and key items 4 were reported as “partially yes.” the remaining key items reporting “yes” are shown: 9 (13/86.7%), 11 (15/100%), 13 (15/100%), 15 (11/73.3%), 15 (11/73.3%), and 15 (11/73.3%). Among the non-critical items, 0 studies reported items 3 and 10, and the remaining non-key items reported “yes” are shown: 5 (14/93.3%), 6 (9/60%), 8 (86.7%), 12 (15/100%), 14 (15/100%), and 16 (1/6.7%), which indicates that the overall quality of the included literature is low. The AMSTAR 2 evaluation is shown in Figure 3 and Table S1, Supplemental Digital Content, http://links.lww.com/MD/K974.

### 3.3. Risk of bias assessment for inclusion in studies

The ROBIS tool was used to assess the 15 studies included in the systematic reviews, rating all included literature as low risk in stage one (assessing relevance) of the ROBIS tool. In stage 2, within domain 1: 73.3% (11/15) of the literature with low risk of bias^[30–36,39–41,43]^ and 26.7% (4/15) of the literature with high risk of bias^[37,38,42,44]^; In domain 2, 66.7% (10/15) of the literature with high risk of bias^[30,33–39,41,44]^ and 33.3% (5/15) of the literature with uncertain risk of bias^[31,32,40,42,43]^; In domain 3, 53.3% (8/15) of the literature on low risk of bias,^[30,32,33,35,39–41,43]^ 40% (6/15) of the literature with high risk of bias^[31,36–38,42,44]^ and 6.7% (1/15) of the literature with uncertain risk of bias^[34]^; And in domain 4, 53.3% (8/15) of the literature with high risk of bias^[31,32,35–37,41,42,44]^ and 46.7% (7/15) of the literature with uncertain risk of bias^[30,33,34,38–40,43]^; In Stage 3, 53.3% (8/15) of the literature with high risk of bias^[31,32,35–37,41,42,44]^ and 46.7% (7/15) of the literature with low risk of bias,^[30,33,34,38–40,43]^ as shown in Table 2.

**Table 2:** ROBIS risk of bias evaluation for inclusion in the literature.

Inclusion in the study	Stage 2				Stage 3
	Domain 1	Domain 2	Domain 3	Domain 4	Risk of bias in the review
Zhang et al 2023^[30]^	Low risk	High risk	Low risk	Uncertain risk	Low risk
Li et al 2021^[31]^	Low risk	Uncertain risk	High risk	High risk	High risk
Liu et al 2021^[32]^	Low risk	Uncertain risk	Low risk	High risk	High risk
Mo et al 2021^[33]^	Low risk	High risk	Low risk	Uncertain risk	Low risk
Liu et al 2020^[34]^	Low risk	High risk	Uncertain risk	Uncertain risk	Low risk
Ji et al 2016^[35]^	Low risk	High risk	Low risk	High risk	High risk
Sun et al 2014^[36]^	Low risk	High risk	High risk	High risk	High risk
Tian. 2019^[37]^	High risk	High risk	High risk	High risk	High risk
Zhu et al 2017^[38]^	High risk	High risk	High risk	Uncertain risk	Low risk
Dou et al 2022^[39]^	Low risk	High risk	Low risk	Uncertain risk	Low risk
Gao et al 2019^[40]^	Low risk	Uncertain risk	Low risk	Uncertain risk	Low risk
Bai et al 2013^[41]^	Low risk	High risk	Low risk	High risk	High risk
Zang et al 2021^[42]^	High risk	Uncertain risk	High risk	High risk	High risk
Chu et al 2022^[43]^	Low risk	Uncertain risk	Low risk	Uncertain risk	Low risk
Liu et al 2017^[44]^	High risk	High risk	High risk	High risk	High risk

All studies were low risk at Stage 1. Stage 2; Domain 1: Study inclusion exclusion criteria; Domain 2: Study search and screening; Domain 3: Data extraction and quality evaluation; Domain 4: Data results and synthetic presentation.

### 3.4. PRISMA quality assessment of included studies

**Figure 4.**
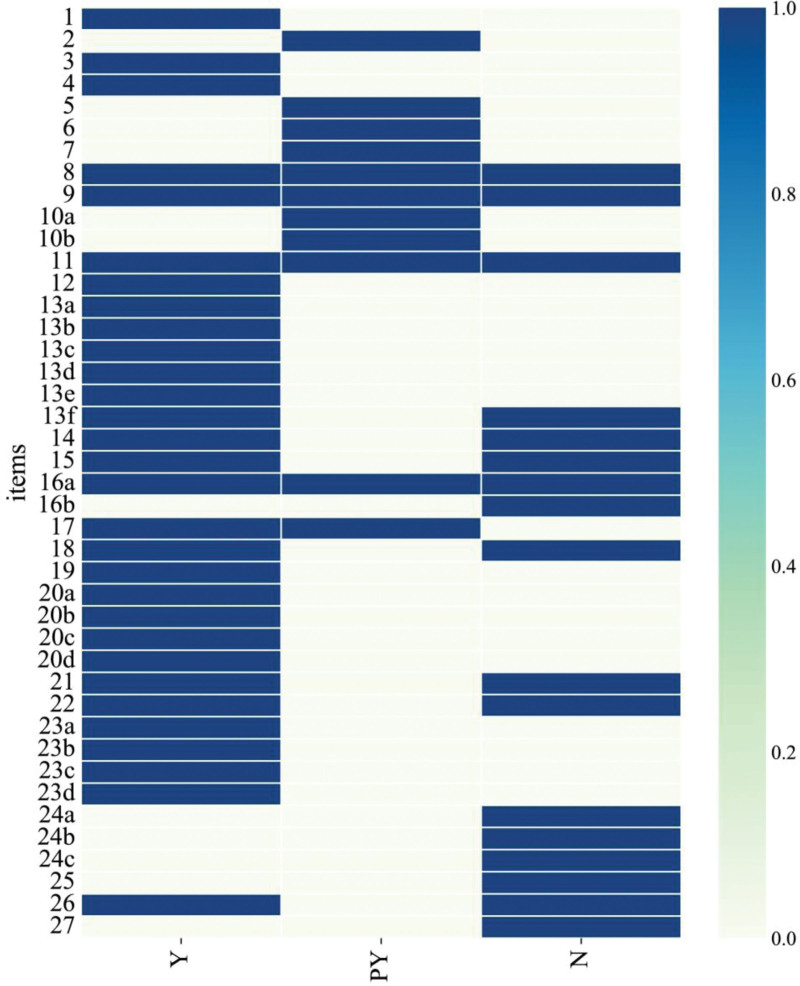
PRISMA 2020 quality assessment incorporated into the systematic evaluation. Note: Y: yes; N: no; PY: partially yes. 1: Title; 2: Abstract; 3: Theoretical basis; 4: Objectives; 5: Eligibility criteria; 6: Information sources; 7: Search strategy; 8: Selection process; 9: Data collection process; 10 (10a.10b): Data items; 11: Assessment of bias risk in individual studies; 12: Effect index; 13 (13a.13b.13c.13d.13e.13f): Synthesis of results; 14: Reporting bias assessment; 15: Methods of outcome index; 16 (16a.16b): Study selection; 17: Characteristics of study; 18: Risk of internal bias in studies; 19: Results of individual studies; 20 (20a.20b.20c.20d): Synthesis of results; 21: Risk of bias between studies; 22: Quality classification of outcome index; 23 (23a.23b.23c.23d): Summary of evidence; 24 (24a.24b.24c): Registration and protocol; 25: Financial support; 26: Declaration of competing interests; 27: Public information.

Evaluating the quality of reporting of included studies using the PRISMA 2020 statement, the methodological quality of 15 included studies^[30–44]^ were evaluated. The results showed that the title, theoretical basis, objectives, effect index, results of individual studies, risk of bias between studies and summary of evidence of the 15 included studies were completely reported. Methods of outcome index, registration and protocol, financial support, declaration of competing interests, public information were rarely reported. Other content of PRISMA 2020 statement were partially reported, as shown in Figure 4 and Table S2, Supplemental Digital Content, http://links.lww.com/MD/K975.

### 3.5. Assessment of the quality of evidence from the included studies

The quality of evidence was assessed for 15 studies, one of which did not synthesize the data, so the evidence quality assessment of 14 studies was carried out. The GRADE 3.6 system tool was used to grade the evidence quality of the 71 outcome indicators included in the literature (Table S3, Supplemental Digital Content, http://links.lww.com/MD/K976). and the outcome indicators were shown to have no high quality. Among them 14 indicators of evidence with moderate quality, accounting for 19.72%; 31 indicators of evidence with low quality, accounting for 43.66%; 26 indicators of evidence with very low quality, accounting for 36.62%. The systematic reviews of literature had a serious risk of bias in the methodologies of the original RCTs studies, with major flaws in randomization, allocation concealment and blinding, which were the main reasons for the downgrading of the quality of evidence in the systematic reviews of studies, followed by publication bias (52, 73.24%), imprecision (11, 15.49%) and inconsistency (24, 33.80%).

#### 3.5.1. GRADE quality assessment of effective rate/total effective rate.

The results of 14^[30–40,42–44]^ studies on systematic reviews of clinical effective rate/total effective rate showed that the use of TCM or TCM combined with Western medicine was more effective in terms of clinical effective rate/total effective rate than conventional Western medicine treatment alone, with a total of 1^[37]^ studies using TCM or TCM combined with Western medicine being rated as evidence with moderate quality and the effect sizes are OR = 3.03 (1.90, 4.84), respectively; One^[43]^ studies with the use of TCM combined with Western medicine were rated as evidence with moderate quality and the effect sizes are RR = 1.20 (1.13, 1.26). These further confirmation the effectiveness of using TCM or TCM combined with Western medicine to treat CB. The evaluation results are shown in Table 3.

**Table 3:** Effective rate/total effective rate comprehensive effect value, and GRADE evidence quality grading.

Outcome indicators	Study	Intervention measures		Number of RCTs (T/C)	Model	OR/RR	95% CI	*I*^2^/%	Downgrade factor	Evidence quality
		T	C							
Effective rate/total effective	Zhang et al 2023^[30]^	TCM	WM	2 (81/81)	Fixed	9.31	2.63, 32.92	0%	②⑦	○○ Low
		TCM+WM	WM	6 (202/202)	Fixed	5.81	2.79, 12.31	0%	②⑦	○○ Low
	Li et al 2021^[31]^	TCM+WM	WM	5 (267/268)	Fixed	1.19	1.12, 1.27	14%	②⑦	○○ Low
	Liu et al 2021^[32]^	TCM+WM	WM	17 (761/756)	Fixed	1.21	1.16, 1.26	0%	②⑦	○○ Low
	Mo et al 2021^[33]^	TCM+WM	WM	48 (2187/2169)	Fixed	−1.21	1.18, 1.24	0%	②⑦	○○ Low
	Liu et al 2020^[34]^	TCM	WM	2 (126/66)	Random	9.88	4.09, 23.88	56%	②③⑤⑦	○○○ Very low
		TCM+WM	WM	10 (444/452)	Fixed	4.65	3.05, 7.09	0%	②⑤⑦	Very low
	Tian. 2019^[37]^	TCM/TCM+WM	TCM/WM	6 (329/283)	Fixed	3.03	1.90, 4.84	0%	②	○ Moderate
	Dou et al 2022^[39]^	TCM+WM	WM	8 (340/340)	Fixed	1.20	1.13, 1.28	0%	②⑦	○○ Low
	Gao et al 2019^[40]^	TCM+WM	WM	14 (577/570)	Fixed	1.19	1.14, 1.23	0%	②⑦	○○ Low
	Zang et al 2021^[42]^	TCM+WM	WM	14 (577/570)	Fixed	4.75	3.23, 7.00	0%	②⑦	○○ Low
	Chu et al 2022^[43]^	TCM+WM	WM	10 (478/478)	Fixed	1.20	1.13, 1.26	0%	②	○ Moderate
	Liu et al 2017^[44]^	TCM+WM	WM	13 (633/616)	Fixed	5.72	3.83, 8.56	0%	②⑦	○○ Low

RCTs = randomized controlled trials, TCM = traditional Chinese medicine, WM = Western medicine.

① most of the information is from studies with high risk of bias, with large flaws in randomization methods, allocation concealment or blinding or without risk of bias assessment; ② some flaws in randomization methods, allocation concealment or blinding; ③ large heterogeneity included in the studies; ④ large heterogeneity included in the studies and without heterogeneity analysis; ⑤ insufficient sample size or wide confidence intervals; ⑥ insufficient sample size and wide confidence intervals; ⑦ left-right asymmetry in the funnel plot.

#### 3.5.2. GRADE quality assessment of disappearance time of clinical symptoms and physical signs.

The results of the 7 studies^[31,32,35,40,42–44]^ included in the systematic reviews showed that the test group can effectively shorten the disappearance time of clinical symptoms and physical signs compared with the control group. It contains a total of 4 moderate -quality items, including the disappearance time of cough and phlegm symptoms, the disappearance time of wheezing symptoms, the disappearance time of rale, and the time of the defervescence. The effects were MD = −0.66 (−0.92, −0.39), MD = −1.09 (−1.54, −0.65), MD = −1.23 (−1.52, −0.95), MD = −0.63 (−0.64, −0.65), MD = −1.23 (−1.52, −0.95), MD = −0.63 (−0.64, −0.65), MD = −1.23 (−1.52, −0.95), MD = −0.63 (−0.96, −0.30), suggesting that the use of TCM or integration of Chinese medicine with Western medicine for treatment is conducive to reducing CB symptoms and shortening the course of CB. The evaluation results are shown in Table 4.

**Table 4:** Comprehensive effect values of disappearance time of clinical symptoms and physical signs, and grading results of GRADE evidence.

Outcome indicators	Study	Intervention measures		Number of RCTs (T/C)	Model	MD	95% CI	*I*^2^/%	Downgrade factor	Evidence quality
		T	C							
Li et al 2021^[31]^	Dyspnea symptom disappearance time	CMT+WM	WM	2 (127/127)	Fixed	−2.68	−3.05, −2.31	0%	②⑦	○○ Low
	Wheezing disappearance time	CMT+WM	WM	2 (127/127)	Fixed	−3.09	−3.48, −2.70	0%	②⑦	○○ Low
	Cough and phlegm disappearance time	CMT+WM	WM	2 (127/127)	Fixed	−3.21	−3.54, −2.09	0%	②⑦	○○ Low
Liu et al 2021^[32]^	Cough disappearance/remission time	CMT+WM	WM	3 (107/103)	Random	−3.13	−4.43, −1.83	76%	②③⑦	○○○ Very low
	Cough and phlegm disappearance time	CMT+WM	WM	3 (107/103)	Fixed	−3.41	−3.99, −2.82	26%	②⑦	○○ Low
	Chest inflammation disappearance time	CMT+WM	WM	2 (92/88)	Fixed	−0.71	−1.71, 0.28	0%	②③⑦	○○○ Very low
Ji et al 2016^[35]^	Clinical symptom disappearance/remission time	CMT	WM	4 (178/176)	Random	5.52	1.99, 15.27	62%	②③⑦	○○○ Very low
Gao et al 2019^[40]^	Cough disappearance/remission time	CMT+WM	WM	6 (368/372)	Random	−2.37	−3.42, −1.32	95%	②③⑦	○○○ Very low
	Antipyretic time	CMT+WM	WM	3 (162/165)	Random	−1.03	−1.45, −0.62	53%	②③⑦	○○○ Very low
	Cough disappearance/remission time	CMT+WM	WM	4 (253/256)	Random	−2.61	−3.63, −1.59	97%	②③⑦	○○○ Very low
Zang et al 2021^[42]^	Cough disappearance/remission time	CMT+WM	WM	6 (218/212)	Random	−2.25	−3.31, −1.20	92%	②③⑦	○○○ Very low
	Cough and phlegm disappearance time	CMT+WM	WM	5 (186/180)	Random	−2.11	−2.91, −1.31	82%	②③⑦	○○○ Very low
	Wheezing symptom disappearance/remission time	CMT+WM	WM	4 (139/139)	Random	−0.96	−1.62, −0.31	87%	②③⑦	○○○ Very low
Chu et al 2022^[43]^	Time for cough symptoms to disappear	CMT+WM	WM	5 (253/253)	Random	−1.08	−1.66, −0.51	68%	②③	○○ Low
	Cough and phlegm disappearance time	CMT+WM	WM	6 (309/309)	Fixed	−0.66	−0.92, −0.39	49%	②	○ Moderate
	Wheezing symptom disappearance/remission time	CMT+WM	WM	4 (159/159)	Fixed	−1.09	−1.54, −0.65	0%	②	○ Moderate
	Longyin disappearance time	CMT+WM	WM	5 (291/291)	Fixed	−1.23	−1.52, −0.95	0%	②	○ Moderate
	Antipyretic time	CMT+WM	WM	3 (168/168)	Fixed	−0.63	−0.96, −0.30	0%	②	○ Moderate
Liu et al 2017^[44]^	Cough disappearance/remission time	CMT+WM	WM	6 (306/298)	Random	−2.04	−3.02, −1.06	99%	②③⑦	○○○ Very low
	Wheezing symptom disappearance/remission time	CMT+WM	WM	5 (276/268)	Fixed	−1.89	−2.07, −1.70	9%	②⑦	○○ Low
	Cough and phlegm disappearance time	CMT+WM	WM	6 (306/299)	Random	−1.60	−3.20, 0.00	100%	②③⑦	○○○ Very low

RCTs = randomized controlled trials, TCM = Traditional Chinese Medicine, WM = Western medicine.

① most of the information is from studies with high risk of bias, with large flaws in randomization methods, allocation concealment or blinding or without risk of bias assessment; ② some flaws in randomization methods, allocation concealment or blinding; ③ large heterogeneity included in the studies; ④ large heterogeneity included in the studies and without heterogeneity analysis; ⑤ insufficient sample size or wide confidence intervals; ⑥ insufficient sample size and wide confidence intervals; ⑦ left-right asymmetry in the funnel plot.

#### 3.5.3. GRADE quality assessment of serum inflammatory factor level.

The results of the 4 studies^[32–34,39]^ included in the systematic reviews showed that TCM combined with Western medicine for treatment can significantly reduce serum inflammatory factor levels, including hypersensitive C-reactive protein, IL-6, IL-8, TNF-α, CRP, white blood cell, and neutrophil levels. One systematic reviews^[43]^ result showed that the test group can significantly reduce the levels of CRP, PCT, TNF-α, and IL-8 compared with the control group, and the difference of white blood cell between the test group and the control group was not statistically significant. It contains 2 evidence items^[43]^ with moderate quality, the effect sizes are: TNF-α: MD = −6.27 (−8.67, −3.87), PCT: MD = −0.57 (−0.62, −0.53) respectively; the results suggest that TCM combined with Western medicine can better reduce the level of CB inflammatory factors. The evaluation results are shown in Table 5.

**Table 5:** Comprehensive effect value of serum inflammatory factor levelsm, and GRADE evidence quality grading.

Outcome indicators	Study	Intervention measures		Number of RCTs (T/C)	Model	MD	95% CI	*I*^2^/%	Downgrade factor	Evidence quality
		T	C							
Liu et al 2021^[32]^	Hs-crp	CMT+WM	WM	2 (71/71)	Random	−12.90	−22.11, −3.69	98%	②③⑤⑦	○○○ Very low
	IL-6	CMT+WM	WM	4 (207/207)	Random	−190.80	−197.61, −183.99	83%	②③⑦	○○○ Very low
	IL-8	CMT+WM	WM	2 (71/71)	Fixed	−76.83	−88.37, −65.28	0%	②③⑤⑦	○○○ Very low
	TNF-α	CMT+WM	WM	6 (278/278)	Random	−87.10	−138.30, −35.91	100%	②③⑤⑦	○○○ Very low
Mo et al 2021^[33]^	CRP	CMT+WM	WM	4 (218/221)	Fixed	−5.67	−6.02, −5.32	9%	②③⑤⑦	○○○ Very low
Liu et al 2020^[34]^	WBC	CMT+WM	WM	2 (95/95)	Fixed	−1.02	−1.35, −0.68	0%	②⑦	○○ Low
	NEUT	CMT+WM	WM	2 (95/95)	Fixed	−4.16	−5.39, −2.92	53%	②③⑦	○○○ Very low
Dou et al 2022^[39]^	CRP	CMT+WM	WM	3 (200/200)	Fixed	−7.32	−8.42, −6.22	0%	②⑦	○○ Low
	IL-8	CMT+WM	WM	2 (104/104)	Random	−63.39	−73.49, −53.29	92%	②③⑦	○○○ Very low
	TNF-α	CMT+WM	WM	4 (200/200)	Fixed	−7.44	−8.35, −6.53	23%	②⑦	○○ Low
Chu et al 2022^[40]^	CRP	CMT+WM	WM	5 (218/218)	Random	−6.06	−9.44, −2.68	94%	②③	○○ Low
	TNF-α	CMT+WM	WM	5 (290/290)	Random	−6.27	−8.67, −3.87	0%	②	○ Moderate
	PCT	CMT+WM	WM	2 (66/66)	Random	−0.57	−0.62, −0.53	0%	②	○ Moderate
	WBC	CMT+WM	WM	2 (101/101)	Fixed	0.48	−0.70, 1.66	58%	②③	○○ Low
	IL-8	CMT+WM	WM	2 (104/104)	Random	−63.51	−73.62, −53.4	94%	②③	○○ Low

RCTs = randomized controlled trials, TCM = traditional Chinese medicine; WM = Western medicine.

① most of the information is from studies with high risk of bias, with large flaws in randomization methods, allocation concealment or blinding or without risk of bias assessment; ② some flaws in randomization methods, allocation concealment or blinding; ③ large heterogeneity included in the studies; ④ large heterogeneity included in the studies and without heterogeneity analysis; ⑤ insufficient sample size or wide confidence intervals; ⑥ insufficient sample size and wide confidence intervals; ⑦ left-right asymmetry in the funnel plot.

#### 3.5.4. Grade quality assessment of lung function indicators and blood gas analysis indicators.

The results of the 4 studies^[31,34,39,43]^ included in the systematic reviews showed that the improvement rate of lung function and indicators of blood gas analysis in the test group was higher than that of the control group. Among them, 5 studies^[43]^ were with moderate-quality evidence, and the effect sizes were FEV1: MD = 0.33 (0.22, 0.43); FVC: MD = 0.32 (0.20, 0.44); FEV1/FVC: MD = 10.17 (8.33, 12.01); peak expiratory flow: MD = 0.70 (0.35, 0.43); FEV1/FVC: MD = 10.17 (8.33, 12.01); peak expiratory flow: MD = 0.70 (0.35, 1.05); arterial oxygen partial pressure: MD = 5.23 (3.20, 7.26) respectively, indicating that TCM combined with Western medicine for treatment can effectively improve lung function. The evaluation results are shown in Table 6.

**Table 6:** Comprehensive effect values of lung function indicators and blood gas analysis, and GRADE grading results.

Outcome indicators	Study	Intervention measures		Number of RCTs (T/C)	Model	MD	95% CI	*I*^2^/%	Downgrade factor	Evidence quality
		T	C							
Li et al 2021^[31]^	Lung function	CMT+WM	WM	3 (198/199)	Fixed	278.72	229.63, 327.80	0%	②⑦	○○ Low
Liu et al 2020^[34]^	FEV1/FVC	CMT+WM	WM	2 (95/95)	Fixed	7.93	5.68, 10.18	90%	②③⑦	○○○ Very low
Dou et al 2022^[39]^	FEV1	CMT+WM	WM	3 (121/121)	Fixed	0.33	0.22, 0.45	0%	②⑦	○○ Low
	FEV1/FVC	CMT+WM	WM	2 (77/77)	Fixed	10.17	8.15, 12.19	0%	②⑦	○○ Low
Chu et al 2022^[43]^	FEV1	CMT+WM	WM	4 (169/169)	Fixed	0.33	0.22, 0.43	0%	②	○ Moderate
	FVC	CMT+WM	WM	4 (169/169)	Fixed	0.32	0.20, 0.44	0%	②	○ Moderate
	FEV1/FVC	CMT+WM	WM	2 (377/77)	Fixed	10.17	8.33, 12.01	0%	②	○ Moderate
	PEF	CMT+WM	WM	2 (92/92)	Fixed	0.70	0.35, 1.05	0%	②	○ Moderate
	PaO2	CMT+WM	WM	2 (92/92)	Fixed	5.23	3.20, 7.26	0%	②	○ Moderate

RCTs = randomized controlled trials, TCM = traditional Chinese medicine, WM = Western medicine.

① most of the information is from studies with high risk of bias, with large flaws in randomization methods, allocation concealment or blinding or without risk of bias assessment; ② some flaws in randomization methods, allocation concealment or blinding; ③ large heterogeneity included in the studies; ④ large heterogeneity included in the studies and without heterogeneity analysis; ⑤ insufficient sample size or wide confidence intervals; ⑥ insufficient sample size and wide confidence intervals; ⑦ left-right asymmetry in the funnel plot.

## 4. Discussion

Reevaluation of systematic reviews is mainly a comprehensive evaluation or a comprehensive analysis through meta-analysis for reevaluation of the etiology, diagnosis, efficacy and safety of the same disease or the same health problem at the current stage, and a method to comprehensively and objectively evaluate the quality and evidence of the included literature through using relevant quality assessment tools. This paper mainly focuses on the literature related to the systematic reviews of TCM treatment of CB published from 2013 to 2023, and through multiple databases or search engines based on the inclusion of exclusion screened a total of 15 pieces of literature involving 224 studies, including 20,710 patients, which ensured an adequate sample size of the study, while strictly following the AMSTAR 2 scale, the PRISMA tool, ROBIS tool and other assessment tools to evaluate the literature, which ensured the reliability of the results of this study. This study mainly reevaluates on the basis of the systematic reviews of CB treatment with TCM, aiming to clarify the authenticity and reliability of the results of previous systematic evaluations, to provide theoretical support for clinicians’ therapeutic decision-making, and at the same time, to provide a reliable reference basis for the formulation of relevant policies and guidelines.^[45,46]^

Based on the AMSTAR 2 quality assessment, all 15 studies were rated with very low quality. The main shortcomings were: the lack of upfront study protocol design and advance registration of information in all studies is not conducive to improving the transparency and credibility of the studies and may lead to selective reporting bias, which in turn affects the rigour of the systematic reviews and increases the risk of bias in the evaluation process; all studies did not provide a justification for the inclusion of the literature, and a detailed screening list of literature would help to reduce the risk of bias, and authors should provide reasons for inclusion; all systematic reviews did not describe the list of studies and items excluded from the literature, and AMSTAR2 scoring items require matching the research question to the PICO of the included literature and describing the reasons for exclusion. The literature included in this study only described the reasons, and did not list the excluded literature in detail; all of the literature did not mention the funding sources and conflicts of interest of individual studies, which did not help readers to determine whether financial sponsorship has an impact on systematic evaluation, and makes systematic reviews suffer from objectivity factors. There are certain defects in the use of risk of bias assessment tools. 26.7% (4/15) of the literature used the JADAD scale to assess the risk of bias of the included studies, but the JADAD score is not assessed for allocation concealment, which may cause incomplete risk of bias assessment information.

The ROBIS evaluation revealed that within Phase 2, in Domain 1, none of the 15 studies included provided a systematic reviews plan, and 11 of these studies described the PICO principles in more detail in the information and methods section, which can be considered as low risk because their principles were probably followed when the systematic reviews was produced. Within Phase 2, in Domain 2, most studies had major deficiencies in searching the literature, such as lack of searches of English databases or clinical registry platforms and failure to conduct searches other than databases. In domain 3, a more detailed presentation of the original literature data is important in order to enhance the credibility of the quantitative synthetic effect values, and some studies were not described in detail; consistent with the AMSTAR 2 assessment, the assessment tool of risk of bias of the original study had deficiencies and should be assessed using a recognized tool such as the Cochrane assessment tool of risk of bias. In domain 4, some studies which did not perform sensitivity analyses or clearly state and discuss the risk of bias of the original study was rated with high risk. In Stage 3, most studies did not discuss all the risk of bias that emerged in Stage 2 in the results section. As the ROBIS tool allows for an objective assessment of the risk of bias in the development of the systematic reviews and in the interpretation of the results, and also assesses the relevance of the systematic reviews to the original studies included, the risk of bias in the systematic reviews can be minimized by conducting a self-assessment in accordance with the items in the ROBIS tool when conducting the systematic reviews.

The 2020 PRISMA statement provides a basis upon which to enhance the quality of systematic reviews. The statement can also define and guide the writing process and the generation of a flow chart representing the systematic review process, and explain relevant items in detail. Nearly half of the 15 systematic reviews included in this study reported significant missing information for their entries. None of the studies gave a list of literature that met the inclusion criteria but was excluded and the reasons for this, did not provide registration information and advice whether a proposal was available, did not advise the source of funding for the study, and the vast majority of studies did not declare conflicts of interest in systematic reviews, did not report publicly available information and provide access (e.g. templates of data extraction form and data from included studies), and did not describe the methods used to evaluate the quality of evidence for each outcome. At the same time, there are varying degrees of missing information in the presentation of structured abstracts, sources of information, search strategies, information entries, and conducting or discussing risk of bias in the study. And all these deficiencies are the reason why the current systematic reviews is generally of low quality and does not serve as a high-quality guidance tool.

The main outcome indicators included in the systematic reviews of this study showed that almost all TCM or TCM combined with Western medicine had definite efficacy on CB. However, the results of the evaluation through the GRADE system showed that 80.28% (57/71) of the 71 outcome indicators included in the systematic reviews were of low or very low quality of evidence, which suggests that the quality of evidence for the outcome indicators of the CB systematic evaluation of TCM treatment at the present stage is low, and that the conclusions of the MAs/SRs may be quite different from the real situation. The main factors contributing to the downgrading of the evidence quality include limitations of the studies. There were certain flaws in the methodological design of the original studies, such as incomplete implementation of blinding, lack of allocation concealment, and selective reporting, and a few studies showed flaws in follow-up bias and reporting bias, which may give rise to additional risks of misleading results. Publication bias. All of the studies included in this study had incomplete searches, which caused a poor symmetry in the funnel plot of systematic reviews. Inconsistency. 33.08% (24/71) of the outcome indicators have little or no overlap in their credibility intervals, and the *P* value obtained by the heterogeneity test is very small and *I*^2^ > 50%, which are obvious significant inconsistencies, the reason for this result may be related to factors such as treatment dose, treatment period, and different outcome measurement tools. However, even when the design and implementation of a systematic reviews are perfect, biased results may still be obtained due to lagged publication^[47]^ and implicit duplicate publication,^[48,49]^ leading to a decline in the quality of the evidence. The methodological limitations of the original studies greatly affect the quality of the evidence evaluated systematically and may lead to biased results. It is recommended that the quality of RCTs should be controlled by following the uniform standards of CONSORT^[50]^ to conduct relevant clinical randomized controlled trials in the future. Imprecision: 15.49% (11/71) of the outcome indicators were also at greater risk of being precise due to wide confidence intervals. The reason for this result is mainly related to the small sample size included, and the small sample size of the original study itself can cause the combined effect size to be too small or the heterogeneity to be too large, both of which can affect the credibility of the results.

In this study, the outcome indicators of 15 included research literature were evaluated, and the results showed that the outcome indicators of treatment with TCM or TCM combined with Western medicine were better than those of the control group. The outcome evaluation indexes were mainly reflected in the clinical effective rate/total effective rate of 73.33% (11/15), serum inflammatory factor level of 46.67% (7/15), time to disappearance of clinical symptoms and signs of 33.33% (5/15), lung function indexes and blood gas analysis indexes of 26.67% (4/15), and the other ones were the coughing shortness of breath score, chest tightness and shortness of breath score, phlegm white adhesion integral, TCM evidence integral, etc. Choosing appropriate outcome evaluation indexes can maximize the real effect of interventions, reduce the cost of the study and improve the efficiency of the study. The establishment of core outcome indicators and the standardization of criteria can improve the quality and transparency of the reporting of MAs/SRs, and also has great practical significance and research significance.

## 5. Limitations

This study is the first to reevaluate the systematic reviews of TCM for the treatment of CB in terms of methodological quality, reporting quality, risk of bias and quality of evidence using the AMSTAR 2 scale, PRISMA tool, ROBIS tool and GRADE system. The evaluation process is relatively transparent and provides evidence to support the use of TCM for the treatment of CB. However, this study has certain limitations, as described below: the study was conducted through a search of published Chinese and English literature, but the search of some gray literature may have resulted in missing, leading to an incomplete search, which may pose a risk of selection bias to the results; the included systematic reviews and its original study did not mention whether patients with CB were identified, so it was not possible to determine whether they were symptomatic treatment. The treatment schemes of the different control groups in the included systematic reviews were uniformly categorized as conventional Western medicine treatment, and there was some heterogeneity among studies in terms of interventions; the reliability and completeness of the conclusions were influenced by the included systematic reviews, which was time-limited; the reevaluation method of this study was influenced by personal subjective judgment, which may have deviated from the real situation bias; most of the studies lacked follow-up data to evaluate the long-term efficacy of the drugs.

## 6. Conclusion

In summary, based on a reevaluation of published MAs/SRs of using TCM for CB treatment, the results show that TCM can exert clinical efficacy by reducing the level of serum inflammatory factors in CB patients, improving the clinical efficiency/total efficiency of treatment, shortening the time for clinical symptoms and signs to disappear, and increasing lung function indicators and blood gas analysis indicators. At the same time, a number of studies have confirmed that TCM has the unique advantage of less adverse reactions in the treatment of CB. However, the associated MAs/SRs were of low methodological quality, with a high overall risk of bias, serious reporting missing and lower quality of evidence. Therefore, in subsequent studies, the relevant specifications of clinical trials should be strictly followed for experimental design, implementation and reporting, such as RCT studies should develop a true randomized sequence, clear allocation of protocol concealment, double-blinding of implementers and participants, blinded measurement of outcome indicators, complete reporting of outcome indicators, etc., in order to reduce the various biases that may arise during the implementation of clinical studies. Investigators of systematic reviews should also strictly follow the appropriate standards and tools to carry out scientific, standardized and rigorous systematic reviews, so as to provide a higher quality decision basis for clinical treatment decisions.

## Author contributions

**Data curation:** Yasheng Deng, Lanhua Xi, Siyin Han, Tianwei Liang, Hui Huang, Yanping Fan, Yiqing Zheng.

**Funding acquisition:** Jiang Lin.

**Investigation:** Yasheng Deng, Hui Huang.

**Writing – original draft:** Yasheng Deng.

**Writing – review & editing:** Jiang Lin.

## Supplementary Material






