# Supplementary material for: Re-evaluation for systematic reviews of traditional Chinese medicine in the treatment of chronic bronchitis
Source: Medicine (Baltimore). 2023 Dec 8;102(49):e36472. doi: 10.1097/MD.0000000000036472 (PMC10713115; doi:10.1097/MD.0000000000036472)
Supplement: Supplementary file 1 [file medi-102-e36472-s001.docx]

**Suppl. Table 1: AMSTAR 2 Methodological quality assessment.**

| Items | Q1 | Q2 | Q3 | Q4 | Q5 | Q6 | Q7 | Q8 | Q9 | Q10 | Q11 | Q12 | Q13 | Q14 | Q15 | Q16 | Score | Quality grade |
| --- | --- | --- | --- | --- | --- | --- | --- | --- | --- | --- | --- | --- | --- | --- | --- | --- | --- | --- |
| Zhang et al. 2023^[30]^ | Y | N | N | PY | N | Y | N | Y | Y | N | Y | Y | Y | Y | Y | N | 9.5 | Very low |
| Li et al. 2021^[31]^ | Y | N | N | PY | Y | N | N | Y | Y | N | Y | Y | Y | Y | N | N | 8.5 | Very low |
| Liu et al. 2021^[32]^ | Y | N | N | PY | Y | Y | N | Y | Y | N | Y | Y | Y | Y | Y | Y | 11.5 | Very low |
| Mo et al. 2021^[33]^ | Y | N | N | PY | Y | Y | N | N | Y | N | Y | Y | Y | Y | Y | N | 9.5 | Very low |
| Liu et al. 2020^[34]^ | Y | N | N | PY | Y | Y | N | Y | Y | N | Y | Y | Y | Y | Y | N | 10.5 | Very low |
| Ji et al. 2016^[35]^ | Y | N | N | PY | Y | Y | N | Y | Y | N | Y | Y | Y | Y | Y | N | 10.5 | Very low |
| Sun et al. 2014^[36]^ | Y | N | N | PY | Y | Y | N | Y | Y | N | Y | Y | Y | Y | N | N | 9.5 | Very low |
| Tian. 2019^[37]^ | Y | N | N | PY | Y | N | N | Y | N | N | Y | Y | Y | Y | Y | N | 8.5 | Very low |
| Zhu et al. 2017^[38]^ | Y | N | N | PY | Y | Y | N | Y | Y | N | Y | Y | Y | Y | Y | N | 10.5 | Very low |
| Dou et al. 2022^[39]^ | Y | N | N | PY | Y | PY | N | N | N | N | Y | Y | Y | Y | N | N | 7 | Very low |
| Gao et al. 2019^[40]^ | Y | N | N | PY | Y | Y | N | Y | Y | N | Y | Y | Y | Y | Y | N | 10.5 | Very low |
| Bai et al. 2013^[41]^ | Y | N | N | PY | Y | PY | N | Y | Y | N | Y | Y | Y | Y | N | N | 9 | Very low |
| Zang et al. 2021^[42]^ | Y | N | N | PY | Y | Y | N | Y | Y | N | Y | Y | Y | Y | Y | N | 10.5 | Very low |
| Chu et al. 2022^[43]^ | Y | N | N | PY | Y | PY | N | Y | Y | N | Y | Y | Y | Y | Y | N | 10 | Very low |
| Liu et al. 2017^[44]^ | Y | N | N | PY | Y | PY | N | Y | Y | N | Y | Y | Y | Y | Y | N | 10 | Very low |

Note: Y: yes; N: no; PY: partially yes. Items: Q1 the detail level of the research questions and inclusion criteria; Q2 the presence of a pre-design programme; Q3 the presence of a rationale for the inclusion of study types; Q4 the comprehensiveness of the search strategy; Q5 whether the literature selection was double-repeat; Q6 whether the data extraction was double-repeat; Q7 the presence of a list of excluded literature and reasons for exclusion; Q8 whether the description of the included studies was detailed; Q9 whether the bias risk was taken with an appropriate tool; Q10 whether the funding source of individual studies was clear; Q11 whether the study results were combined using appropriate statistical methods; Q12 whether the impact of the bias risk of the included studies on the results was assessed; Q13 whether the bias risk of the included studies was considered; Q14 whether the heterogeneity of the study results was assessed; Q15 whether the possibility of publication bias was evaluated; Q16 whether relevant conflicts of interest were reported.
