# Supplementary material for: Re-evaluation for systematic reviews of traditional Chinese medicine in the treatment of chronic bronchitis
Source: Medicine (Baltimore). 2023 Dec 8;102(49):e36472. doi: 10.1097/MD.0000000000036472 (PMC10713115; doi:10.1097/MD.0000000000036472)
Supplement: Supplementary file 2 [file medi-102-e36472-s002.docx]

**Suppl. Table 2: PRISMA 2020 quality evaluation included in the literature.**

| Items | Serial number | Zhang et al. 2023^[30]^ | Li et al. 2021^[31]^ | Liu et al. 2021^[32]^ | Mo et al. 2021^[33]^ | Liu et al. 2020^[34]^ | Ji et al. 2016^[35]^ | Sun et al. 2014^[36]^ | Tian. 2019^[37]^ | Zhu et al. 2017^[38]^ | Dou et al. 2022^[39]^ | Gao et al. 2019^[40]^ | Bai et al. 2013^[41]^ | Zang et al. 2021^[42]^ | Chu et al. 2022^[43]^ | Liu et al. 2017^[44]^ | Y（%） | PY（%） | N（%） |
| --- | --- | --- | --- | --- | --- | --- | --- | --- | --- | --- | --- | --- | --- | --- | --- | --- | --- | --- | --- |
| Title | 1 | Y | Y | Y | Y | Y | Y | Y | Y | Y | Y | Y | Y | Y | Y | Y | 15 | 0 | 0 |
| Abstract | 2 | PY | PY | PY | PY | PY | PY | PY | PY | PY | PY | PY | PY | PY | PY | PY | 0 | 15 | 0 |
| Theoretical basis | 3 | Y | Y | Y | Y | Y | Y | Y | Y | Y | Y | Y | Y | Y | Y | Y | 15 | 0 | 0 |
| Objectives | 4 | Y | Y | Y | Y | Y | Y | Y | Y | Y | Y | Y | Y | Y | Y | Y | 15 | 0 | 0 |
| Eligibility criteria | 5 | PY | PY | PY | PY | PY | PY | PY | PY | PY | PY | PY | PY | PY | PY | PY | 0 | 15 | 0 |
| Information sources | 6 | PY | PY | PY | PY | PY | PY | PY | PY | PY | PY | PY | PY | PY | PY | PY | 0 | 15 | 0 |
| Search strategy | 7 | PY | PY | PY | PY | PY | PY | PY | PY | PY | PY | PY | PY | PY | PY | PY | 0 | 15 | 0 |
| Selection process | 8 | PY | N | Y | Y | N | Y | N | N | Y | Y | Y | Y | Y | Y | N | 9 | 1 | 5 |
| Data collection process | 9 | PY | PY | Y | Y | Y | Y | Y | N | Y | Y | Y | Y | Y | Y | PY | 11 | 3 | 1 |
| Data items | 10a | PY | PY | PY | PY | PY | PY | PY | PY | PY | PY | PY | PY | PY | PY | PY | 0 | 15 | 0 |
|  | 10b | PY | PY | PY | PY | PY | PY | PY | PY | PY | PY | PY | PY | PY | PY | PY | 0 | 15 | 0 |
| Assessment of bias risk in individual studies | 11 | PY | Y | PY | PY | Y | PY | PY | N | PY | Y | Y | N | Y | Y | Y | 7 | 6 | 2 |
| Effect index | 12 | Y | Y | Y | Y | Y | Y | Y | Y | Y | Y | Y | Y | Y | Y | Y | 15 | 0 | 0 |
| Synthesis of results | 13a | Y | Y | Y | Y | Y | Y | Y | Y | Y | Y | Y | Y | Y | Y | Y | 15 | 0 | 0 |
|  | 13b | Y | Y | Y | Y | Y | Y | Y | Y | Y | Y | Y | Y | Y | Y | Y | 15 | 0 | 0 |
|  | 13c | Y | Y | Y | Y | Y | Y | Y | Y | Y | Y | Y | Y | Y | Y | Y | 15 | 0 | 0 |
|  | 13d | Y | Y | Y | Y | Y | Y | Y | Y | Y | Y | Y | Y | Y | Y | Y | 15 | 0 | 0 |
|  | 13e | Y | Y | Y | Y | Y | Y | Y | Y | Y | Y | Y | Y | Y | Y | Y | 15 | 0 | 0 |
|  | 13f | N | N | N | N | N | N | N | N | Y | N | N | N | N | Y | N | 2 | 0 | 13 |
| Reporting bias assessment | 14 | Y | N | Y | Y | Y | Y | Y | Y | Y | N | Y | N | Y | Y | Y | 12 | 0 | 3 |
| Methods of outcome index | 15 | N | N | N | Y | N | N | N | N | N | N | N | N | Y | N | N | 2 | 0 | 13 |
| Study selection | 16a | Y | Y | Y | N | Y | N | N | N | N | PY | Y | N | PY | Y | Y | 7 | 2 | 6 |
|  | 16b | N | N | N | N | N | N | N | N | N | N | N | N | N | N | N | 0 | 0 | 15 |
| Characteristics of study | 17 | Y | Y | Y | PY | Y | PY | Y | Y | Y | Y | Y | Y | Y | Y | Y | 13 | 2 | 0 |
| Risk of internal bias in studies | 18 | Y | Y | Y | Y | Y | Y | Y | Y | N | Y | Y | Y | N | Y | Y | 13 | 0 | 2 |
| Results of individual studies | 19 | Y | Y | Y | Y | Y | Y | Y | Y | Y | Y | Y | Y | Y | Y | Y | 15 | 0 | 0 |
| Synthesis of results | 20a | Y | Y | Y | Y | Y | Y | Y | Y | Y | Y | Y | Y | Y | Y | Y | 15 | 0 | 0 |
|  | 20b | Y | Y | Y | Y | Y | Y | Y | Y | Y | Y | Y | Y | Y | Y | Y | 15 | 0 | 0 |
|  | 20c | Y | Y | N | Y | Y | Y | N | Y | Y | Y | Y | Y | Y | Y | N | 15 | 0 | 0 |
|  | 20d | Y | N | N | Y | Y | N | N | N | Y | Y | Y | N | N | Y | N | 15 | 0 | 0 |
| Risk of bias between studies | 21 | Y | Y | Y | Y | Y | Y | Y | Y | N | Y | Y | Y | Y | Y | Y | 14 | 0 | 1 |
| Quality classification of outcome index | 22 | N | N | N | Y | N | N | N | N | N | N | N | N | Y | N | N | 2 | 0 | 13 |
| Summary of evidence | 23a | Y | Y | Y | Y | Y | Y | Y | Y | Y | Y | Y | Y | Y | Y | Y | 15 | 0 | 0 |
|  | 23b | Y | Y | Y | Y | Y | Y | Y | Y | Y | Y | Y | Y | Y | Y | Y | 15 | 0 | 0 |
|  | 23c | Y | Y | Y | Y | Y | Y | Y | Y | Y | Y | Y | Y | Y | Y | Y | 15 | 0 | 0 |
|  | 23d | Y | Y | Y | Y | Y | Y | Y | Y | Y | Y | Y | Y | Y | Y | Y | 15 | 0 | 0 |
| Registration and protocol | 24a | N | N | N | N | N | N | N | N | N | N | N | N | N | N | N | 0 | 0 | 15 |
|  | 24b | N | N | N | N | N | N | N | N | N | N | N | N | N | N | N | 0 | 0 | 15 |
|  | 24c | N | N | N | N | N | N | N | N | N | N | N | N | N | N | N | 0 | 0 | 15 |
| Financial support | 25 | N | N | N | N | N | N | N | N | N | N | N | N | N | N | N | 0 | 0 | 15 |
| Declaration of competing interests | 26 | N | N | Y | N | N | N | N | N | N | N | N | N | N | N | N | 1 | 0 | 14 |
| Public information | 27 | N | N | N | N | N | N | N | N | N | N | N | N | N | N | N | 0 | 0 | 15 |
|  | PY | 9 | 7 | 7 | 8 | 6 | 8 | 7 | 6 | 7 | 7 | 6 | 6 | 7 | 6 | 7 |  |  |  |
|  | N | 10 | 13 | 11 | 9 | 11 | 12 | 14 | 15 | 12 | 11 | 10 | 14 | 10 | 9 | 13 |  |  |  |
|  | Y | 23 | 22 | 24 | 25 | 25 | 22 | 21 | 21 | 23 | 24 | 26 | 22 | 25 | 27 | 22 |  |  |  |
|  | Total score | 27.5 | 25.5 | 27.5 | 29 | 28 | 26 | 24.5 | 24 | 26.5 | 27.5 | 29 | 25 | 28.5 | 30 | 25.5 |  |  |  |

Note: Y: yes; N: no; PY: partially yes.
