# Supplementary material for: Re-evaluation for systematic reviews of traditional Chinese medicine in the treatment of chronic bronchitis
Source: Medicine (Baltimore). 2023 Dec 8;102(49):e36472. doi: 10.1097/MD.0000000000036472 (PMC10713115; doi:10.1097/MD.0000000000036472)
Supplement: Supplementary file 3 [file medi-102-e36472-s003.docx]

**Suppl. Table 3: The main outcome indicators included in the systematic evaluation GRADE evidence quality grading results.**

| Included studies | Outcome indicators | Intervention measures | | Number of RCTs | Models | Effect size | *P* | *I^2^* | limitations | inconsistency | indirectness | imprecision | publication bias | Evidence quality |
| --- | --- | --- | --- | --- | --- | --- | --- | --- | --- | --- | --- | --- | --- | --- |
|  |  | T | C | (Therapy group/Control) |  |  |  |  |  |  |  |  |  |  |
| Zhang et al. 2023^[30]^ | effective rate | TCM+WM | WM | 2(81/81) | Fixed | OR=9.31,  95%CI(2.63,32.92) | P=0.0005 | 0% | -1^②^ | 0 | 0 | 0 | -1^⑦^ | ○○  Low |
|  |  | TCM+WM | WM | 6(202/202) | Fixed | OR=5.81,  95%CI(2.79,12.31) | P＜0.00001 | 0% | -1^②^ | 0 | 0 | 0 | -1^⑦^ | ○○  Low |
|  | recurrence rate | TCM+WM | WM | 2(80/80) | Fixed | MD=0.16,  95%CI(0.05,0.48) | P=0.001 | 0% | -1^②^ | 0 | 0 | -1^⑤^ | -1^⑦^ | ○○○  Very low |
|  |  | TCM+WM | WM | 4(125/125) | Fixed | MD=0.13,  95%CI(0.05,0.29) | p＜0.00001 | 0% | -1^②^ | 0 | 0 | 0 | -1^⑦^ | ○○  Low |
|  | Cough and shortness of breath points | TCM/TCM+WM | WM | 3(93/93) | Fixed | MD=﹣4.16,  95%CI(﹣4.69,﹣3.64) | p＜0.00001 | 0% | -1^②^ | 0 | 0 | -1^⑤^ | -1^⑦^ | ○○○  Very low |
|  | Chest tightness and shortness of breath points | TCM/TCM+WM | WM | 3(93/93) | Fixed | MD=﹣4.97,  95%CI(﹣5.57,﹣4.37) | p＜0.00001 | 39% | -1^②^ | 0 | 0 | -1^⑤^ | -1^⑦^ | ○○○  Very low |
|  | Phlegm white adhesion points | TCM/TCM+WM | WM | 3(93/93) | Fixed | MD=﹣4.70,  95%CI(﹣5.61,﹣3.79) | p＜0.00001 | 59% | -1^②^ | -1^③^ | 0 | -1^⑤^ | -1^⑦^ | ○○○  Very low |
| Li et al. 2021^[31]^ | total effective rate | TCM+WM | WM | 5(267/268) | Fixed | RR=1.19,  95%CI(1.12,1.27) | p＜0.00001 | 14% | -1^②^ | 0 | 0 | 0 | -1^⑦^ | ○○  Low |
|  | lung function | TCM+WM | WM | 3(198/199) | Fixed | MD=278.72,  95%CI(229.63,327.80) | p＜0.00001 | 0% | -1^②^ | 0 | 0 | 0 | -1^⑦^ | ○○  Low |
|  | dyspnea disappearance time | TCM+WM | WM | 2(127/127) | Fixed | MD=﹣2.68,  95%CI(﹣3.05,﹣2.31) | p＜0.00001 | 0% | -1^②^ | 0 | 0 | 0 | -1^⑦^ | ○○  Low |
|  | wheezing symptom disappearance/remission time | TCM+WM | WM | 2(127/127) | Fixed | MD=﹣3.09,  95%CI(﹣3.48,﹣2.70) | p＜0.00001 | 0% | -1^②^ | 0 | 0 | 0 | -1^⑦^ | ○○  Low |
|  | cough and phlegm disappearance/remission time | TCM+WM | WM | 2(127/127) | Fixed | MD=﹣3.21,  95%CI(﹣3.54,﹣2.09) | p＜0.00001 | 0% | -1^②^ | 0 | 0 | 0 | -1^⑦^ | ○○  Low |
| Liu et al. 2021^[32]^ | effective rate | TCM+WM | WM | 17(761/756） | Fixed | MD=1.21,  95%CI(1.16,1.26) | p＜0.00001 | 0% | -1^②^ | 0 | 0 | 0 | -1^⑦^ | ○○  Low |
|  | cough disappearance/remission time | TCM+WM | WM | 3(107/103) | Random | MD=﹣3.13,  95%CI(﹣4.43,﹣1.83) | p＜0.00001 | 76% | -1^②^ | -1^③^ | 0 | 0 | -1^⑦^ | ○○○  Very low |
|  | cough and phlegm disappearance/remission time | TCM+WM | WM | 3(107/103) | Fixed | MD=﹣3.41,  95%CI(﹣3.99,﹣2.82) | p＜0.00001 | 26% | -1^②^ | 0 | 0 | 0 | -1^⑦^ | ○○  Low |
|  | chest inflammation disappearance time | TCM+WM | WM | 2(92/88) | Fixed | MD=﹣0.71,  95%CI(﹣1.71,0.28) | P=0.16 | 0% | -1^②^ | 0 | 0 | -1^⑤^ | -1^⑦^ | ○○○  Very low |
|  | Hs-crp | TCM+WM | WM | 2(71/71) | Random | MD=﹣12.90,  95%CI(﹣22.11,﹣3.69) | P=0.006 | 98% | -1^②^ | -1^③^ | 0 | -1^⑤^ | -1^⑦^ | ○○○  Very low |
|  | IL-6 | TCM+WM | WM | 4(207/207) | Random | MD=﹣190.80,  95%CI(﹣197.61,﹣183.99) | p＜0.00001 | 83% | -1^②^ | -1^③^ | 0 | 0 | -1^⑦^ | ○○○  Very low |
|  | IL-8 | TCM+WM | WM | 2(71/71) | Fixed | MD=﹣76.83,  95%CI(﹣88.37,﹣65.28) | p＜0.00001 | 0% | -1^②^ | 0 | 0 | -1^⑤^ | -1^⑦^ | ○○○  Very low |
|  | TNF-α | TCM+WM | WM | 6(278/278) | Random | MD=﹣87.10,  95%CI(﹣138.30,﹣35.91) | P=0.0009 | 100% | -1^②^ | -1^③^ | 0 | -1^⑤^ | -1^⑦^ | ○○○  Very low |
| Mo et al. 2021^[33]^ | effective rate | TCM+WM | WM | 48(2187/2169) | Fixed | RR=﹣1.21,  95%CI(1.18,1.24) | p＜0.00001 | 0% | -1^②^ | 0 | 0 | 0 | -1^⑦^ | ○○  Low |
|  | CRP | TCM+WM | WM | 4(218/221) | Fixed | MD=﹣5.67,  95%CI(﹣6.02,﹣5.32) | p＜0.00001 | 4% | -1^②^ | 0 | 0 | -1^⑤^ | -1^⑦^ | ○○○  Very low |
|  | adverse reactions | TCM+WM | WM | 18(810/805) | Random | MD=0.79,  95%CI(0.47,1.34) | P=0.39 | 44% | -1^②^ | 0 | 0 | 0 | -1^⑦^ | ○○  Low |
| Liu et al. 2020^[34]^ | effective rate | TCM | WM | 2(126/66) | Random | OR=9.88,  95%CI(4.09,23.88) | p＜0.00001 | 56% | -1^②^ | -1^③^ | 0 | -1^⑤^ | -1^⑦^ | ○○○  Very low |
|  |  | TCM+WM | WM | 10(444/452) | Fixed | OR=4.65,  95%CI(3.05,7.09) | p＜0.00001 | 0% | -1^②^ | 0 | 0 | -1^⑤^ | -1^⑦^ | ○○○  Very low |
|  | cure rate | TCM/TCM+WM | WM | 8(298/306) | Fixed | MD=2.28,  95%CI(1.57,3.32) | p＜0.0001 | 0% | -1^②^ | 0 | 0 | 0 | -1^⑦^ | ○○  Low |
|  | FEV1/FVC | TCM+WM | WM | 2(95/95) | Fixed | MD=7.93,  95%CI(5.68,10.18) | p＜0.00001 | 90% | -1^②^ | -1^③^ | 0 | 0 | -1^⑦^ | ○○○  Very low |
|  | WBC | TCM+WM | WM | 2(95/95) | Fixed | MD=﹣1.02,  95%CI(﹣1.35,﹣0.68) | p＜0.00001 | 0% | -1^②^ | 0 | 0 | 0 | -1^⑦^ | ○○  Low |
|  | NEUT | TCM+WM | WM | 2(95/95) | Fixed | MD=﹣4.16,  95%CI(﹣5.39,﹣2.92) | p＜0.00001 | 53% | -1^②^ | -1^③^ | 0 | 0 | -1^⑦^ | ○○○  Very low |
| Ji et al. 2016^[35]^ | clinical effects | TCM | WM | 8(446/363) | Fixed | OR=4.61,  95%CI(2.94,7.22) | p＜0.00001 | 0% | -1^②^ | 0 | 0 | 0 | -1^⑦^ | ○○  Low |
|  |  | TCM+WM | WM | 3(115/93) | Fixed | OR=5.52,  95%CI(1.99,15.27) | P=0.001 | 0% | -1^②^ | 0 | 0 | 0 | -1^⑦^ | ○○  Low |
|  | clinical symptom disappearance/remission time | TCM | WM | 4(178/176) | Random | MD=5.52,  95%CI(1.99,15.27) | p＜0.00001 | 62% | -1^②^ | -1^③^ | 0 | 0 | -1^⑦^ | ○○○  Very low |
|  | hospitalization time | TCM | WM | 4(178/176) | Random | MD=5.52,  95%CI(1.99,15.27) | p＜0.00001 | 57% | -1^②^ | -1^③^ | 0 | 0 | -1^⑦^ | ○○○  Very low |
| Sun et al. 2014^[36]^ | clinical effects | TCM | WM | 13(798/758) | Fixed | OR=6.62,  95%CI(4.58,9.58) | p＜0.00001 | 0% | -1^②^ | 0 | 0 | 0 | -1^⑦^ | ○○  Low |
| Tian. 2019^[37]^ | effective rate | TCM/TCM+WM | TCM/WM | 6(329/283) | Fixed | OR=3.03,  95%CI(1.90,4.84) | p＜0.00001 | 0% | -1^②^ | 0 | 0 | 0 | 0 | ○  Moderate |
|  | TCM syndrome points | TCM/TCM+WM | TCM/WM | 6(329/283) | Random | MD=2.67,  95%CI(1.61,3.73) | p＜0.00001 | 67% | -1^②^ | -1^③^ | 0 | 0 | 0 | ○○  Low |
| Zhu et al. 2017^[38]^ | clinical effects | TCM+WM | WM | 19(1003/993) | Random | OR=4.72,  95%CI(3.5,6.36) | p＜0.00001 | 0% | -1^②^ | 0 | 0 | 0 | 0 | ○  Moderate |
| Dou et al. 2022^[39]^ | effective rate | TCM+WM | WM | 8(340/340) | Fixed | RR=1.20,  95%CI(1.13，1.28) | p＜0.00001 | 0% | -1^②^ | 0 | 0 | 0 | -1^⑦^ | ○○  Low |
|  | CRP | TCM+WM | WM | 3(200/200) | Fixed | MD=﹣7.32,  95%CI(﹣8.42,﹣6.22) | p＜0.00001 | 0% | -1^②^ | 0 | 0 | 0 | -1^⑦^ | ○○  Low |
|  | （FEV1） | TCM+WM | WM | 3(121/121) | Fixed | MD=0.33,  95%CI(0.22,0.45) | p＜0.00001 | 0% | -1^②^ | 0 | 0 | 0 | -1^⑦^ | ○○  Low |
|  | （FEV1/FVC） | TCM+WM | WM | 2(77/77) | Fixed | MD=10.17,  95%CI(8.15,12.19) | p＜0.00001 | 0% | -1^②^ | 0 | 0 | 0 | -1^⑦^ | ○○  Low |
|  | IL-8 | TCM+WM | WM | 2(104/104) | Random | MD=﹣63.39,  95%CI(﹣73.49,﹣53.29) | p＜0.00001 | 92% | -1^②^ | -1^③^ | 0 | 0 | -1^⑦^ | ○○○  Very low |
|  | TNF-α | TCM+WM | WM | 4(200/200) | Fixed | MD=﹣7.44,  95%CI(﹣8.35,﹣6.53) | p＜0.00001 | 23% | -1^②^ | 0 | 0 | 0 | -1^⑦^ | ○○  Low |
| Gao et al. 2019^[40]^ | cough disappearance/remission time | TCM+WM | WM | 6(368/372) | Random | MD=﹣2.37,  95%CI(﹣3.42,﹣1.32) | p＜0.00001 | 95% | -1^②^ | -1^③^ | 0 | 0 | -1^⑦^ | ○○○  Very low |
|  | antipyretic time | TCM+WM | WM | 3(162/165) | Random | MD=﹣1.03,  95%CI(﹣1.45,﹣0.62) | p＜0.00001 | 53% | -1^②^ | -1^③^ | 0 | 0 | -1^⑦^ | ○○○  Very low |
|  | cough disappearance/remission time | TCM+WM | WM | 4(253/256) | Random | MD=﹣2.61,  95%CI(﹣3.63,﹣1.59) | p＜0.00001 | 97% | -1^②^ | -1^③^ | 0 | 0 | -1^⑦^ | ○○○  Very low |
|  | total effective rate | TCM+WM | WM | 23(952/949) | Random | RR=1.19,  95%CI(1.14,1.23) | p＜0.00001 | 0% | -1^②^ | 0 | 0 | 0 | -1^⑦^ | ○○  Low |
| Zang et al. 2021^[42]^ | effective rate | TCM+WM | WM | 14(577/570) | Fixed | OR=4.75,  95%CI(3.23,7.00) | p＜0.00001 | 0% | -1^②^ | 0 | 0 | 0 | -1^⑦^ | ○○  Low |
|  | cough disappearance/remission time | TCM+WM | WM | 6(218/212) | Random | MD=﹣2.25,  95%CI(﹣3.31,﹣1.20) | p＜0.00001 | 92% | -1^②^ | -1^③^ | 0 | 0 | -1^⑦^ | ○○○  Very low |
|  | cough and phlegm disappearance/remission time | TCM+WM | WM | 5(186/180) | Random | MD=﹣2.11,  95%CI(﹣2.91,﹣1.31) | p＜0.00001 | 82% | -1^②^ | -1^③^ | 0 | 0 | -1^⑦^ | ○○○  Very low |
|  | wheezing symptom disappearance/remission time | TCM+WM | WM | 4(139/139) | Random | MD=﹣0.96,  95%CI(﹣1.62,﹣0.31) | P=0.004 | 87% | -1^②^ | -1^③^ | 0 | 0 | -1^⑦^ | ○○○  Very low |
| Chu et al. 2022^[43]^ | effective rate | TCM+WM | WM | 10(478/478) | Fixed | RR=1.20,  95%CI(1.13,1.26) | p＜0.00001 | 0% | -1^②^ | 0 | 0 | 0 | 0 | ○  Moderate |
|  | cough disappearance/remission time | TCM+WM | WM | 5(253/253) | Random | MD=﹣1.08,  95%CI(﹣1.66,﹣0.51) | P=0.0002 | 68% | -1^②^ | -1^③^ | 0 | 0 | 0 | ○○  Low |
|  | cough and phlegm disappearance/remission time | TCM+WM | WM | 6(309/309) | Fixed | MD=﹣0.66,  95%CI(﹣0.92,﹣0.39) | p＜0.00001 | 49% | -1^②^ | 0 | 0 | 0 | 0 | ○  Moderate |
|  | wheezing symptom disappearance/remission time | TCM+WM | WM | 4(159/159) | Fixed | MD=﹣1.09,  95%CI(﹣1.54,﹣0.65) | p＜0.00001 | 0% | -1^②^ | 0 | 0 | 0 | 0 | ○  Moderate |
|  | Rales disappearance time | TCM+WM | WM | 5(291/291) | Fixed | MD=﹣1.23,  95%CI(﹣1.52,﹣0.95) | p＜0.00001 | 0% | -1^②^ | 0 | 0 | 0 | 0 | ○  Moderate |
|  | antipyretic time | TCM+WM | WM | 3(168/168) | Fixed | MD=﹣0.63,  95%CI(﹣0.96,﹣0.30) | P=0.0002 | 0% | -1^②^ | 0 | 0 | 0 | 0 | ○  Moderate |
|  | FEV1 | TCM+WM | WM | 4(169/169) | Fixed | MD=0.33,  95%CI(0.22,0.43) | p＜0.00001 | 0% | -1^②^ | 0 | 0 | 0 | 0 | ○  Moderate |
|  | FVC | TCM+WM | WM | 4(169/169) | Fixed | MD=0.32,  95%CI(0.20,0.44) | p＜0.00001 | 0% | -1^②^ | 0 | 0 | 0 | 0 | ○  Moderate |
|  | FEV1/FVC | TCM+WM | WM | 2(377/77) | Fixed | MD=10.17,  95%CI(8.33,12.01) | p＜0.00001 | 0% | -1^②^ | 0 | 0 | 0 | 0 | ○  Moderate |
|  | PEF | TCM+WM | WM | 2(92/92) | Fixed | MD=0.70,  95%CI(0.35,1.05) | p＜0.0001 | 0% | -1^②^ | 0 | 0 | 0 | 0 | ○  Moderate |
|  | PaO2 | TCM+WM | WM | 2(92/92) | Fixed | MD=5.23,  95%CI(3.20,7.26) | p＜0.00001 | 0% | -1^②^ | 0 | 0 | 0 | 0 | ○  Moderate |
|  | CRP | TCM+WM | WM | 5(218/218) | Random | MD=﹣6.06,  95%CI(﹣9.44,﹣2.68) | P=0.0004 | 94% | -1^②^ | -1^③^ | 0 | 0 | 0 | ○○  Low |
|  | TNF-α | TCM+WM | WM | 5(290/290) | Random | MD=﹣6.27,  95%CI(﹣8.67,﹣3.87) | p＜0.00001 | 0% | -1^②^ | 0 | 0 | 0 | 0 | ○  Moderate |
|  | PCT | TCM+WM | WM | 2(66/66) | Random | MD=﹣0.57,  95%CI(﹣0.62,﹣0.53) | p＜0.00001 | 0% | -1^②^ | 0 | 0 | 0 | 0 | ○  Moderate |
|  | WBC | TCM+WM | WM | 2(101/101) | Fixed | MD=0.48,  95%CI(﹣0.70,1.66) | P=0.43 | 58% | -1^②^ | -1^③^ | 0 | 0 | 0 | ○○  Low |
|  | IL-8 | TCM+WM | WM | 2(104/104) | Random | MD=﹣63.51,  95%CI(﹣73.62,﹣53.4) | p＜0.00001 | 94% | -1^②^ | -1^③^ | 0 | 0 | 0 | ○○  Low |
| Liu et al. 2017^[44]^ | total effective rate | TCM+WM | WM | 13(633/616) | Fixed | OR=5.72,  95%CI(3.83,8.56) | p＜0.00001 | 0% | -1^②^ | 0 | 0 | 0 | -1^⑦^ | ○○  Low |
|  | cough disappearance/remission time | TCM+WM | WM | 6(306/298) | Random | MD=﹣2.04,  95%CI(﹣3.02,﹣1.06) | p＜0.00001 | 99% | -1^②^ | -1^③^ | 0 | 0 | -1^⑦^ | ○○○  Very low |
|  | wheezing symptom disappearance/remission time | TCM+WM | WM | 5(276/268) | Fixed | MD=﹣1.89,  95%CI(﹣2.07,﹣1.70) | p＜0.00001 | 9% | -1^②^ | 0 | 0 | 0 | -1^⑦^ | ○○  Low |
|  | cough and phlegm disappearance/remission time | TCM+WM | WM | 6(306/299) | Random | MD=﹣1.60,  95%CI(﹣3.20,0.00) | P=0.05 | 100% | -1^②^ | -1^③^ | 0 | 0 | -1^⑦^ | ○○○  Very low |

**Note:** TCM: Traditional Chinese Medicine; WM: Western medicine. ① most of the information is from studies with high risk of bias, with large flaws in randomisation methods, allocation concealment or blinding or without risk of bias assessment; ② some flaws in randomisation methods, allocation concealment or blinding; ③ large heterogeneity included in the studies; ④ large heterogeneity included in the studies and without heterogeneity analysis; ⑤ insufficient sample size or wide confidence intervals; ⑥ insufficient sample size and wide confidence intervals; ⑦ left-right asymmetry in the funnel plot.
